# Quantitative performance of photon-counting CT at low dose: Virtual monochromatic imaging and iodine quantification

**DOI:** 10.1002/mp.16583

**Published:** 2023-07-06

**Authors:** Stevan Vrbaski, Steve Bache, Jayasai Rajagopal, Ehsan Samei

**Affiliations:** 1 Department of Radiology, Carl E. Ravin Advanced Imaging Laboratories, Duke University Medical Center, Durham, North Carolina, USA; 2 Department of Physics, University of Trieste, Trieste, Italy; 3 Elettra-Sincrotrone Trieste, Basovizza, Trieste, Italy; 4 Clinical Imaging Physics Group, Department of Radiology, Duke University Medical Center, Durham, North Carolina, USA; 5 Radiology and Imaging Sciences,Clinical Center, National Institutes of Health, Bethesda, Maryland, USA

**Keywords:** iodine quantification, photon-counting CT, virtual monochromatic imaging

## Abstract

**Background:**

Quantitative imaging techniques, such as virtual monochromatic imaging (VMI) and iodine quantification (IQ), have proven valuable diagnostic methods in several specific clinical tasks such as tumor and tissue differentiation. Recently, a new generation of computed tomography (CT) scanners equipped with photon-counting detectors (PCD) has reached clinical status.

**Purpose:**

This work aimed to investigate the performance of a new photon-counting CT (PC-CT) in low-dose quantitative imaging tasks, comparing it to an earlier generation CT scanner with an energy-integrating detector dual-energy CT (DE-CT).The accuracy and precision of the quantification across size, dose, material types (including low and high iodine concentrations),displacement from iso-center, and solvent (tissue background) composition were explored.

**Methods:**

Quantitative analysis was performed on two clinical scanners, Siemens SOMATOM Force and NAEOTOM Alpha using a multi-energy phantom with plastic inserts mimicking different iodine concentrations and tissue types. The tube configurations in the dual-energy scanner were 80/150Sn kVp and 100/150Sn kVp, while for PC-CT both tube voltages were set to either 120 or 140 kVp with photon-counting energy thresholds set at 20/65 or 20/70 keV. The statistical significance of patient-related parameters in quantitative measurements was examined using ANOVA and pairwise comparison with the posthoc Tukey honest significance test. Scanner bias was assessed in both quantitative tasks for relevant patient-specific parameters.

**Results:**

The accuracy of IQ and VMI in the PC-CT was comparable between standard and low radiation doses (*p* < 0.01). The patient size and tissue type significantly affect the accuracy of both quantitative imaging tasks in both scanners. The PC-CT scanner outperforms the DE-CT scanner in the IQ task in all cases. Iodine quantification bias in the PC-CT (−0.9 ± 0.15 mg/mL) at low doses in our study was comparable to that of DE-CT (range −2.6 to 1.5 mg/mL, published elsewhere) at a 1.7× higher dose, but the dose reduction severely biased DE-CT (4.72 ± 0.22 mg/mL). The accuracy in Hounsfield units (HU) estimation was comparable for 70 and 100 keV virtual imaging between scanners, but PC-CT was significantly underestimating virtual 40 keV HU values of dense materials in the phantom representing the extremely obese population.

**Conclusions:**

The statistical analysis of our measurements reveals better IQ at lower radiation doses using new PC-CT. Although VMI performance was mostly comparable between the scanners, the DE-CT scanner quantitatively outperformed PC-CT when estimating HU values in the specific case of very large phantoms and dense materials, benefiting from increased X-ray tube potentials.

## 1 ∣ INTRODUCTION

Spectral computed tomography (CT) has allowed the possibility of a more quantitative evaluation of data acquired from clinical scanners. The term quantitative imaging is often broadly defined and includes anything from estimating material physical quantities (e.g., density and effective atomic number^1^) to data displaying methods. Virtual monochromatic imaging (VMI) and iodine quantification (IQ) are the major quantitative representations in spectral CT to highlight specific attributes of clinical information. For example, VMI promises to offer improved differentiation of tissues by targeting specific points of their μ(E) dependence, while further correcting for beam-hardening artifacts^2^ with benefits in imaging abdomen,^3^ lungs^4^ and head and neck.^5^ The clinical relevance of IQ has been reflected in many studies. The most significant application includes the studies of pulmonary disease,^6^ coronary artery disease through the detection of myocardial ischemia based on contrast distribution,^7^ tumor status (e.g., thymic epithelial tumor^8^), differentiation of metastatic and non-metastatic lymph nodes,^9^ renal masses and hepatocellular carcinoma,^10-12^ and lung cancer where IQ has significant diagnostic and prognostic power.

Increased popularity and demand for VMI and IQ has resulted in several studies testing their accuracy, precision, and repeatably across different vendors and scanning conditions offering spectral information. Most investigations have focused on the identification of sources of error when estimating quantitative parameters and comparing results from different scanner generations. Jacobsen et al.^13^ performed a study measuring the iodine concentration bias from different scanners and computing VMI data at several energies. Following the suggestion from this paper, Euler et al.^14^ conducted a large study among second and third-generation CT scanners focused on minimum detectable concentration difference under various scan and patient-related factors. Several other studies compared different scanner generations and vendors based on acquisition technique,^7^ iterative reconstruction, tube settings, and patient size,^15^ fluid characteristics of solvent, and influence of the iodine concentration itself.^16^ These studies show that third-generation dual-energy CT (DE-CT) scanners outperform the second-generation models. The difference between single-source fast kV-switching and DE-CT in estimating IQ and VMI maps was found to have a small impact. The choice of reconstruction algorithm (i.e., filtered-back projection or iterative reconstruction) and exposure level showed either insignificant or very minor impact^17,18^ on quantitative measurements. Most of the variability was introduced through non-controllable factors such as patient size, iodine concentration, solvent chemical composition, and misalignment of the phantoms with respect to the iso-center (patient positioning errors).

Recently, a new generation of CT scanners equipped with photon-counting detectors (PCD) has reached clinical status. This started a new era of CT since the process of data collection with PCDs is inherently different from energy-integrating detectors (EID). Images can be obtained with reduced noise while maintaining comparable or better spatial resolution. In other words, photon-counting CT (PC-CT) is capable of delivering images of comparable or better quality at significantly reduced doses. For the computation of virtual monochromatic images and material decomposition (such as IQ), at least two independent sources of information are needed. With clinically available systems with EIDs, this is accomplished with both source-based methods (e.g., dual X-Ray tubes operating at differing tube voltages, differential filtration on a single X-Ray source, or with rapid tube-voltage switching with a single source) and detector-based methods (e.g., multi-layered detectors). In PCD each photon is recorded once its energy is above a certain energy threshold. Spectral separation of detected photons with PCD is therefore enabled during the counting process through the choice of two (or more) energy thresholds. While spectral imaging on earlier scanners is limited by the overlap of low and high-energy spectra, the approach of photon binning in PCD can potentially improve the accuracy of estimated quantities.^19,20^

During its development, the photon-counting approach to detection was extensively studied using simulation frameworks, bench setups, and prototype systems.^21-23^ Rajendran et al.^24^ performed one of the first technical evaluations of a clinical PC-CT scanner, demonstrating improved spatial resolution as well as the potential for lower radiation dose and image noise when compared to current state-of-the-art CT systems. Booij et al.^25^ compared the contrast-to-noise ratio of an iodinated contrast agent in DE-CT and PC-CT at three different phantom sizes and several VMI levels. They demonstrated improved PC-CT performance only at VMI levels below 60 keV. They also discovered that using a tube voltage of 90 kV results in a higher contrast-to-noise ratio (CNR) than using a tube voltage of 120 kV. Sartoretti et al.^26^ conducted a comparison study in 30 patients using the same two scanners for IQ in liver parenchyma and lesions. They demonstrated good IQ accuracy regardless of radiation dose, iodine concentration, or base attenuation. Both studies found no significant differences in PC-CT and DE-CT^26^ or minor differences in iodine-to-tissue CNR at low energy VMIs (40 – 60 keV)^25^ at radiation doses recommended by diagnostic reference levels (DRLs). Decker et al.^27^ published the first low-dose study in clinical PC-CT, demonstrating a statistically significant improvement in CNR and image noise in abdominal scans when compared to a second-generation scanner. This study demonstrated that PCD technology produces higher-quality images at lower doses, but it was not quantitative in the sense that VMI levels were not compared. Liu et al.^28^ provided a quantitative evaluation of multiple VMI levels between DE-CT and PC-CT Siemens scanners at low doses, reporting improved accuracy in a large phantom and a significant reduction in electronic background noise. In both of the low-dose studies, the IQ task was not evaluated.

This work aimed to investigate the potential of PC-CT for low-dose quantitative spectral tasks on the NAEOTOM Alpha, a newly released CT scanner in the clinical environment. The scanner was evaluated against a clinical DE-CT scanner (Siemens Somatom FORCE) in VMI and IQ tasks.The study tested the accuracy of both Siemens scanners in estimating Hounsfield units (HU) and iodine concentrations of phantom inserts against the ground truth. Guided by the evidence from previous research,^18,25^ scan-related parameters were closely matched, shifting the focus to patient-specific and the most influential parameters: phantom volume, material type, its location in the scanner, and also comparing the relevant concentration of iodine (2 mg/mL) in two different tissue backgrounds (solvents) - water and blood. The virtual monochromatic images were generated at energies of 40, 70, and 100 keV. The radiation dose used for image acquisition needed special consideration. Previous research on quantitative imaging in DE-CT scanners indicates optimal performance at current DRLs, but research PC-CT systems showed significant improvement in contrast-to-noise ratio at the same doses.^24,29^ Thus, in addition to routine dose levels, data in this study was collected at doses below the current DRLs, according to the potential of the new technology. Moreover, using the combination of large phantom sizes and extra-low doses we explored the ranges within which quantitative imaging remains viable.

## 2 ∣ MATERIALS AND METHODS

### 2.1 ∣ Phantom

**FIGURE 1:**
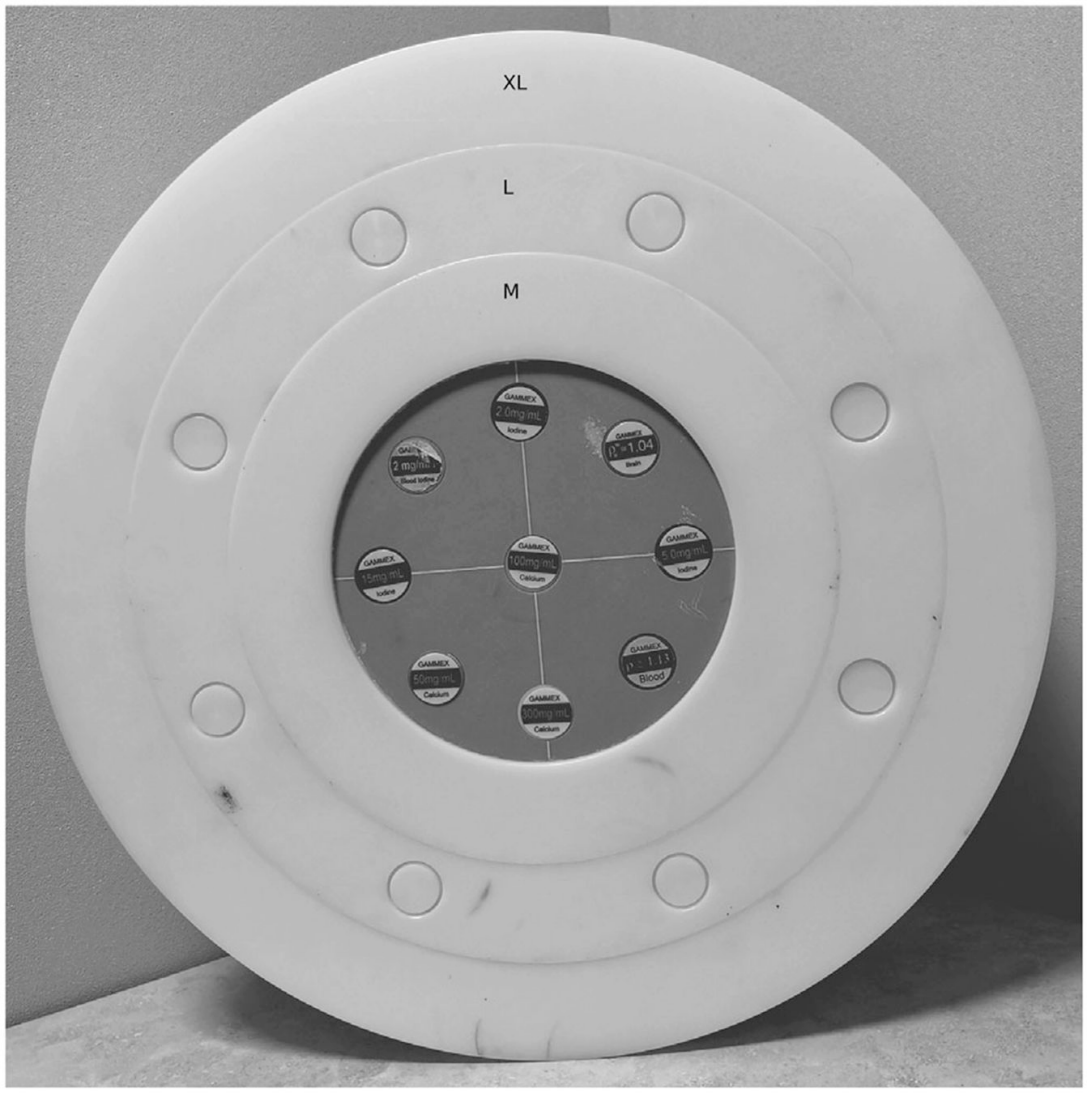
The phantom with inserts used for data collection. The core is a commercial Multi-Energy CT Phantom (Model 1472, Gammex Inc.) while fat-equivalent rings were produced in-house to simulate different patient sizes. CT, computed tomography.

The Multi-Energy 20-cm-diameter CT Phantom (Model 1472, Gammex Inc.) containing nine different inserts that were used for this study is shown in Figure 1. Inserts contained iodine (concentrations of 2, 5, and 15 mg/mL), calcium (50,100, and 300 mg/mL), and body tissues (brain and blood). Custom-made rings (each 5 cm in width) of fat-equivalent material were added to simulate the waist circumference (WC) of larger patients. There were a total of three rings, referred to as M, L, and XL sizes, designed according to the NIH practical guide to represent normal (WC: 90-100 cm), type-I, and type-II obese (WC: 110-130 cm), and extreme obese (WC: > 130 cm) patients.

### 2.2 ∣ CT scanners

Quantitative analysis was performed on two clinical scanners: Siemens FORCE and Siemens NAEOTOM Alpha. The FORCE scanner is representative of a third-generation DU-CT system, utilizing the latest energy-integrating detector technology. Spectral separation is enabled by two X-Ray tubes which are simultaneously operated at different tube voltages. Thus, in spectral mode, the standard outputs of this system are “low” and “high” energy projections. The tube operating at higher energy has also a smaller field of view, limiting the quantitative analysis for very large patients. The NAEOTOM Alpha is a first-generation PC-CT scanner. It is a dual-source scanner with two CdTe PCD with both tubes operating at the same voltage. The in-plane resolution in ultra-high mode reaches 0.125 mm,^24^ somewhat higher than the 0.30 mm in the FORCE scanner. In lower resolution “standard mode”, spectral separation is possible by up to four energy levels, but in high resolution “ultrahigh-resolution mode”, only two energy thresholds are available. It is worth noting that even when operating in standard mode by averaging the values in the 2-by-2 pixel neighborhood,the effective pixel size is still smaller than in the FORCE scanner.The reconstruction software uses an iterative reconstruction approach (Quantum Iterative Reconstruction, or QIR) different from the one that comes with the FORCE scanner (ADMIRE) and the standard output is always a VMI dataset. Sartoretti et al.^18^ showed a reduction of up to 45% in the global noise index between filtered back-projection and maximum QIR. This improvement didn’t compromise the noise texture and mean attenuation values of measured regions. Thus, the iterative strength in the NAEOTOM Alpha scanner has no influence on quantitative values except for measurement standard deviation.

### 2.3 ∣ Acquisition and reconstruction

For each phantom size, we determined the required effective mAs to achieve volume CT dose index (${\mathrm{CTDI}}_{vol}$) values that align with our clinical quality reference mAs (QRM) for a routine abdomen pelvis exam. Given the desire to use the new technology for low-dose imaging, the clinical ${\mathrm{CTDI}}_{vol}$ was then halved and quartered to give three dose levels. In this study, scanners were matched based on ${\mathrm{CTDI}}_{vol}$ values and we refer to dose levels as standard, low, and extra-low doses. In addition, for each dose level, the phantom was shifted 5 cm in a vertical direction to ascertain the effect of variability in patient positioning on the results. The acquisition parameters used on both scanners are given in Table 1. In the DE-CT scanner, the low voltage tube was set to 80 kVp or 100 kVp and high to 150 Sn kVp,^15,22,26^ while for PC-CT both tubes were at 120 or 140 kVp, with the two thresholds set at 20 and 65 keV^30^ and 20 and 70 keV, respectively. All scans were acquired at the pitch of 1.

**FIGURE 2:**
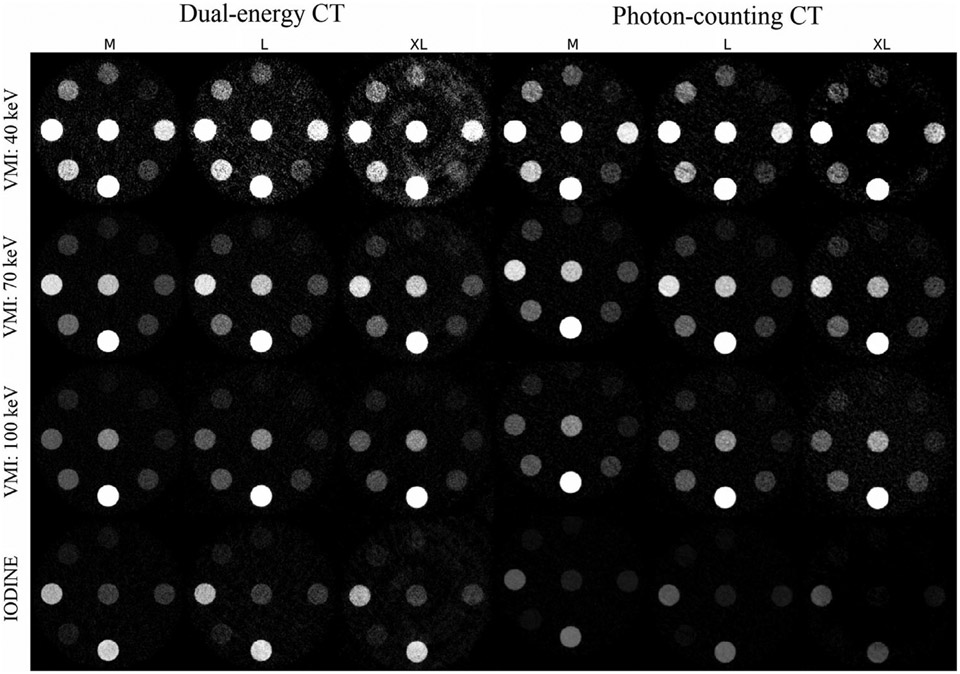
The extra-low dose phantom CT reconstructions from the DE-CT and the PC-CT scanner organized by size (M, L, XL) for three virtual monochromatic energy levels (40, 70, and 100 keV) and an iodine map. Scans are displayed with window width 0–500 HU and the orientation of inserts is the same as in Figure 1. CT, computed tomography; DE-CT, dual-energy CT; HU, Hounsfield units; PC-CT, photon-counting CT.

The quantitative reconstruction kernel “Qr40” was used with both scanners. The influence of iterative reconstruction strength was shown to be negligible in the presence of other patient-related parameters in DE-CT^14,15^ and it also does not bias mean values in PC-CT.^18^ Because QIR has 4 degrees of strength and ADMIRE has 5, we decided to use strength 3 for the NAEOTOM Alpha scanner (75%, Syngo VA40) and strength 4 (80%, Syngo VB10) for the FORCE scanner. All reconstructions were performed using a slice thickness of 2 mm, a field of view of 500 mm, and a matrix size of 512 × 512. The choice of VMI energy level is task-dependent and for non-contrast tasks, 70 keV images are a vendor standard output on NAEOTOM Alpha PC-CT. Besides 70 keV, images at lower energy (40 keV) and higher energy (100 keV) were rendered on both scanners to enable a full comparison. Ground truth values provided by the phantom manufacturer were calculated from the elemental compositions of the inserts. For IQ, measured values exported in DICOM iodine maps were converted to units of iodine concentration (mg/mL)^14^ using vendor calibration. Figure 2 shows extra-low dose scans for qualitative comparison.

### 2.4 ∣ Statistical evaluation

An automated approach to data collection was implemented (Python, version 3.10.0) and statistical analysis was performed in dedicated statistical software (R, version 4.1.3). Pixel values were extracted from the nine circular regions in each slice (16 mm in diameter). A total of 10 slices free from major artifacts were considered for each scan. All data acquired were initially separated into two groups according to scanner type. The association between measured and true values of iodine concentration and HU values across all scanner conditions was statistically evaluated using Pearson correlation. Experimental data were compared to ground truth values across different patient-related parameters using known material compositions provided by the phantom manufacturer. To assess the accuracy of measurements, the difference D = (mean measured value – true value) was computed for each region of interest (ROI) leading to a total of 90 values per scan. An uncertainty on the difference was reported using 95% confidence intervals computed as 1.96 × standard error. Using D as the dependent variable and analysis of variance (ANOVA), the influence of each patient-related parameter (patient size, radiation dose, solvent type, and displacement from the iso-center) on the accuracy of each scanner was assessed. After the most significant sources of error were identified, further analysis was performed using posthoc Tukey Honest Significance Difference ( HSD) on the same dataset. The goal of this step was the pairwise comparison of groups within the selected patient-related parameter to estimate the influence of each group on the accuracy of measurement (D). To consider overall deviation from the ground truth, a virtual monochromatic scanner bias was estimated as:

(1) $$MB=\sum_{i=40,\;70,\;100\;keV}{\left(\text{measured}-\text{true}\right)}_{i}$$

and an iodine scanner bias was defined as:

(2) $$IB=\sum_{i=2,\;5,\;15\;mg∕mL}{\left(\text{measured}-\text{true}\right)}_{i}$$

previously defined by Jacobsen et al.^13^ The results reported in this study were obtained at a *p* < 0.01 statistical significance level.

## 3 ∣ RESULTS

### 3.1 ∣ Quantitative assessment of virtual monochromatic data

**FIGURE 3:**
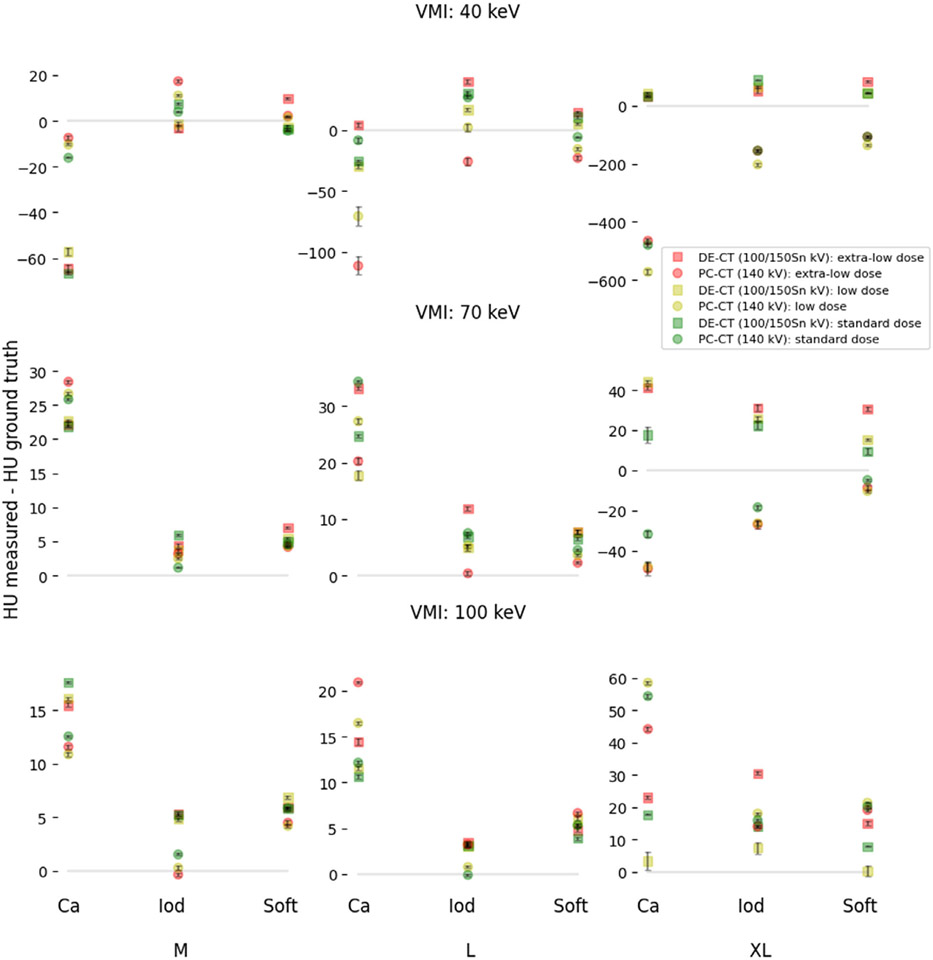
Comparison of the three virtual monochromatic levels (40, 70, and 100 keV) as deviations from the ground truth (*baseline equals no deviation*), for a combination of three patient-related factors (phantom size, dose, and material density), highlighting the difference between PC-CT and DE-CT. The data points represent the mean differences obtained from 10 different slices, while the error bars are the mean standard errors. Similar tissue inserts (soft, calcium, iodine), and values measured when the phantom was displaced from the iso-center were averaged together. Data points were grouped based on phantom size (M, L, XL), radiation dose (extra-low: red, low: yellow, standard: green), and material insert type. DE-CT, dual-energy CT; PC-CT, photon-counting CT.

In our analysis, we obtained superior HU and IQ accuracy for higher tube power setups (100/150Sn kVp and 140 kVp) in large and extra-large phantoms at low doses, and a comparable accuracy in the medium-size phantom. Thus, subsequent analysis was performed using higher voltage setups, and the raw data visualization for major patient-specific dependencies is shown in Figure 3. The results of the Pearson test showed a statistically significant correlation between measured and the phantom manufacturer (true) HU values in both scanners. A slightly higher correlation coefficient of 0.993 was observed for the DE-CT scanner, versus 0.970 for the PC-CT scanner. The overall mean difference with 95% confidence interval for the combined contribution of all patient-related factors was 13 ± 2 HU for DE-CT and −28 ± 3 HU for PC-CT at all three VMI energy levels, 14 ± 4 and −83 ± 6 for 40 keV, 17 ± 1 and −1 ± 1 for 70 keV, and 10 ± 1 and 14 ± 1 for 100 keV, for DE-CT and PC-CT, respectively.

The analysis of variance revealed that the choice of monochromatic energy level in both scanners had a significant effect on the difference between actual and measured values. All patient-related parameters used in this study significantly affected the accuracy of HU values in the DE-CT, while in the PC-CT scanner, the radiation dose and the displacement proved insignificant for the VMI in tested conditions. The ANOVA results for VMI are shown in the first part of Table 2.

**FIGURE 4:**
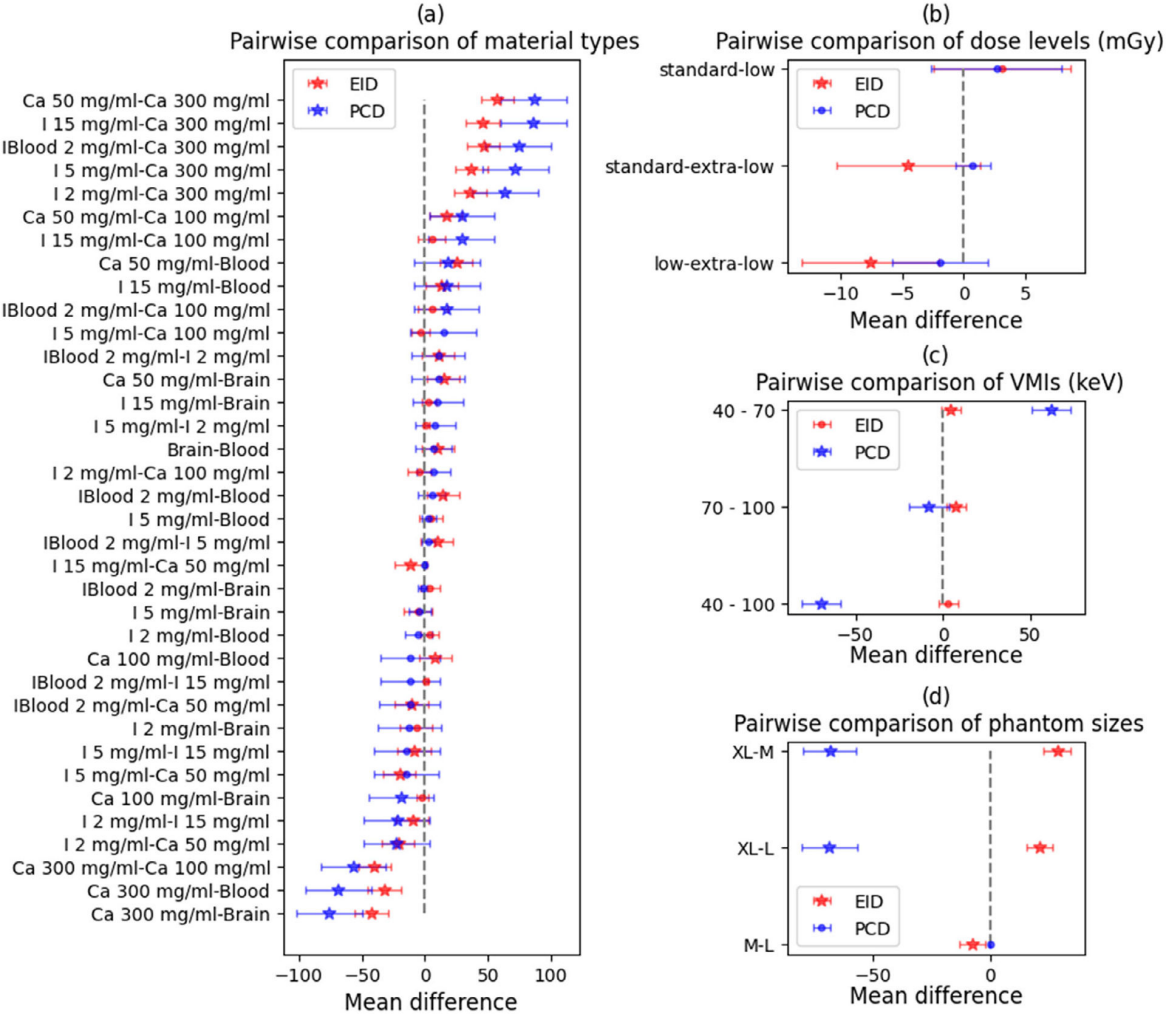
Tukey pairwise comparisons of groups within each statistically significant patient-related parameter for the VMI task are presented with 95% family-wise confidence intervals. Panels show a comparison of mean differences of accuracy (D) between material types (a), dose levels (b), VMI levels (c), and phantom sizes (d). Star shapes (★) mark the statistically significant mean differences of accuracy (D), while circles show insignificant pairwise mean differences. VMI, virtual monochromatic imaging.

The posthoc Tukey pairwise comparison of the significant patient-related parameters is summarized in Figure 4. The statistically significant difference in the pairwise comparison of dense material inserts containing calcium and high iodine concentrations against other materials was observed in both scanners (Figure 4a). The most obvious difference between the two scanners was observed in a pairwise comparison of dose levels: in the PC-CT scanner, there were no statistically significant differences between dose levels while in DE-CT significant differences were observed in the comparison of extra-low dose levels and the other two higher dose levels (Figure 4b). The VMI levels significantly differed from each other, especially in PC-CT where the average accuracy D for 40 keV VMI level and XL phantom was −503 HU in calcium, −182 in iodine, and −117 in soft inserts (given in more detail in the Appendix). The mean difference between all sizes in both scanners was statistically significant, but a major increase in mean difference was associated with the extra-large phantom size.

**FIGURE 5:**
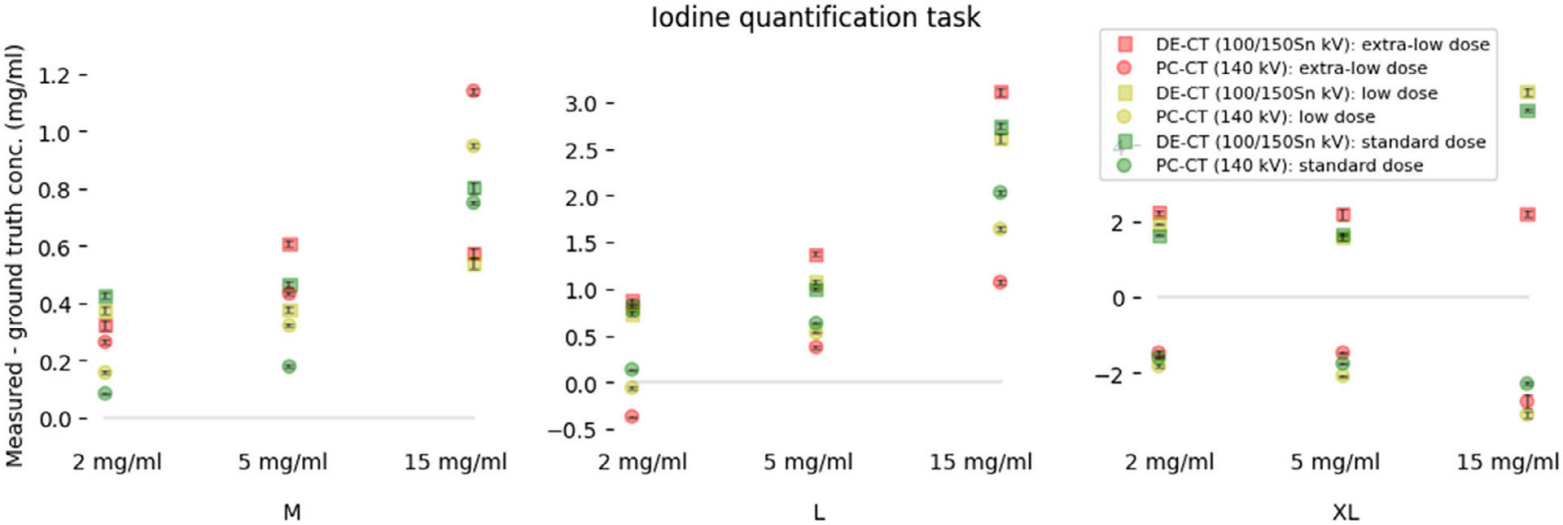
Comparison of measured iodine concentrations to the ground truth, highlighting the difference between PC-CT and DE-CT for respective tube configurations 140 kVp and 100/150Sn kVp. The data points represent the mean differences obtained from 10 different slices, while the error bars are the mean standard errors. All data points were grouped by phantom size (M, L, XL), radiation dose, and level of iodine concentration (2, 5, and 15 mg/mL). Iodine inserts at 2 mg/mL for two different tissue backgrounds (water and blood), and values measured when the phantom was displaced from the iso-center were averaged together. DE-CT, dual-energy CT; PC-CT, photon-counting CT.

The monochromatic bias was characterized for each VMI level according to the formulation given in Section 2.4 and shown later in Figure 5. The results demonstrated the opposite overall MB of 40 ± 5 HUs in DE-CT and −83 ± 7 in the PC-CT scanner. A major contribution to a negative bias in the PC-CT scanner was driven by the underestimation of HU values at 40 keV virtual monochromatic images, especially in dense phantom inserts (Ca 300 mg/mL and iodine 15 mg/mL).

### 3.2 ∣ Quantitative assessment of IQ

**FIGURE 6:**
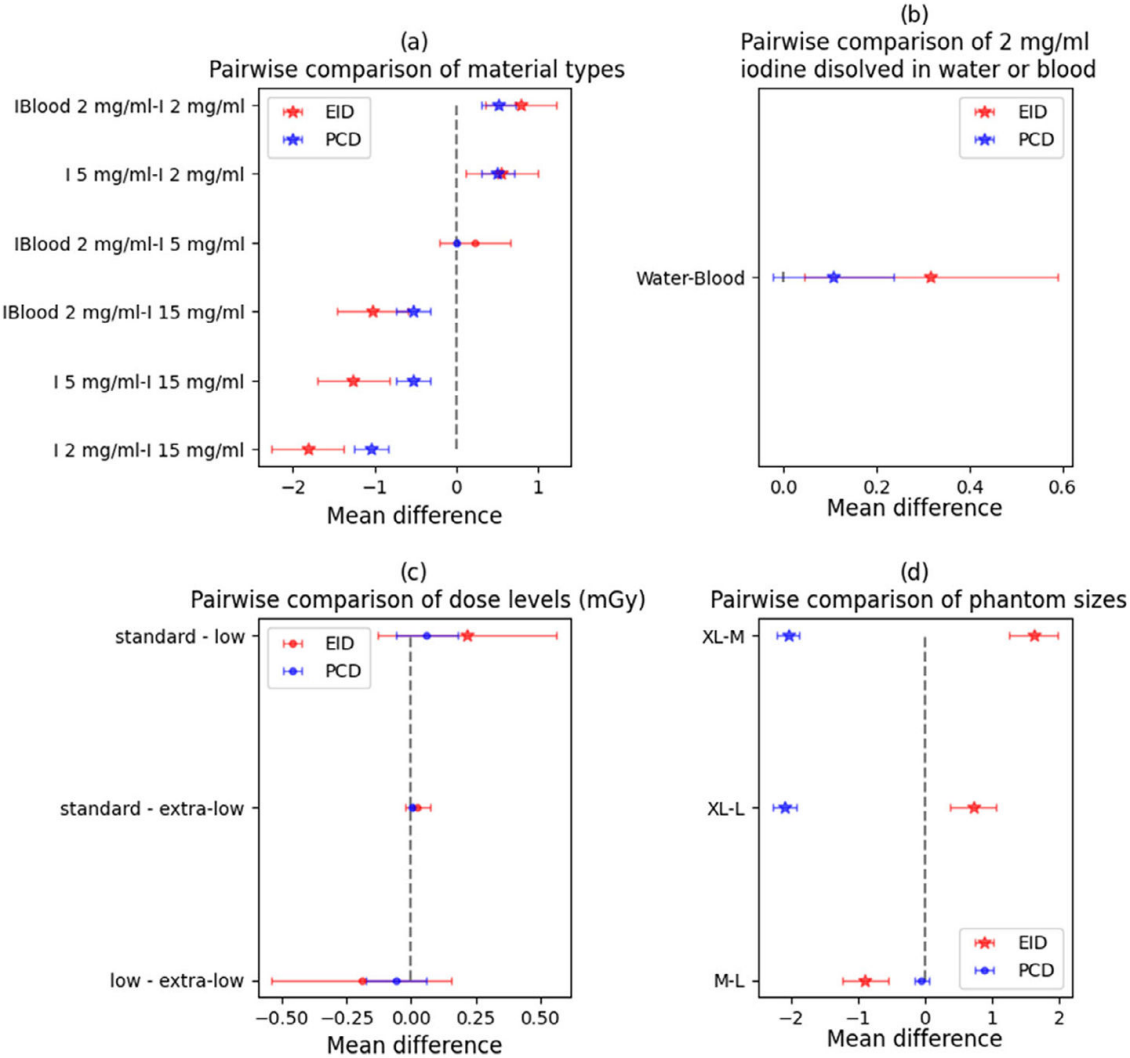
Tukey pairwise comparisons of elements within each statistically significant patient-related parameter for the IQ task are presented with 95% family-wise confidence intervals. Panels show a comparison of mean differences of accuracy (D) between iodine concentration (a), tissue backgrounds (b), dose levels (c), and phantom sizes (d). Star shapes (★) mark the statistically significant differences in the observed mean differences. IQ, iodine quantification.

Measured iodine concentrations showed a statistically significant correlation with ground truth values in both scanners for the task of IQ. The Pearson correlation in PC-CT (0.89) was significantly higher than in DE-CT (0.80). Visual inspection of the data revealed that patient-related parameters have a lower influence on the accuracy and stability of measurements in the PC-CT scanner. For the IQ task, a comparison between the two scanners is shown in Figure 6. Data were sorted by the level of iodine concentration, radiation dose, and phantom size.

**FIGURE 7:**
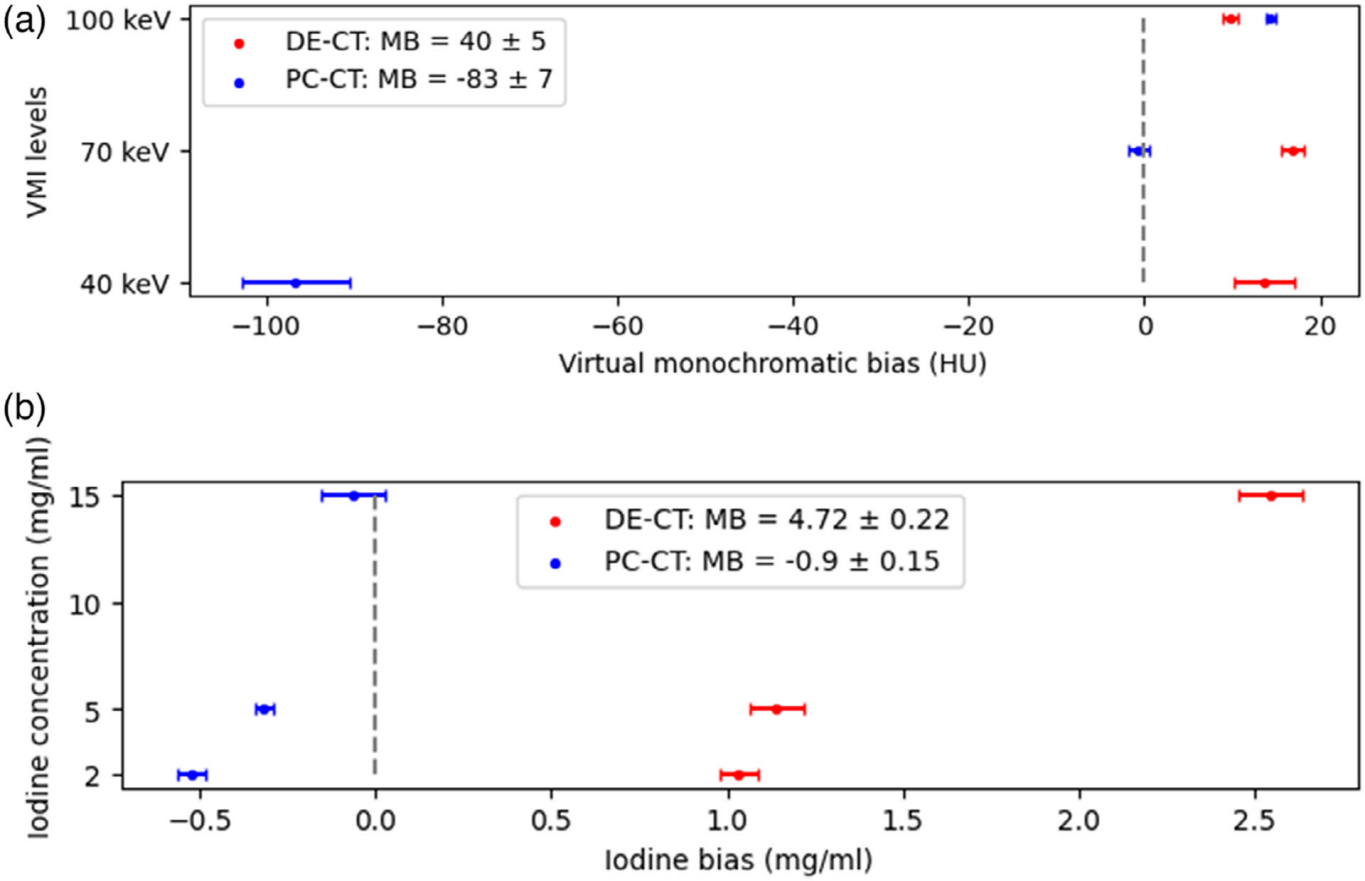
Scanner bias: VMI and iodine bias computed using Equations (1) and (2), respectively. The horizontal lines show the 95% confidence intervals. VMI, Virtual monochromatic imaging.

The overall mean difference when all patient-related factors influencing the IQ task were taken into account was 1.57 ± 0.04 mg/mL in the DE-CT and −0.30 ± 0.02 mg/mL in the PC-CT, with significant differences between DE-CT and PC-CT. The mean difference increased with the phantom size, 0.50 ± 0.03 and 0.47 ± 0.01 for M size, 1.59 ± 0.06 and 0.67 ± 0.03 for L size, and 2.63 ± 0.13 and −2.04 ± 0.11 for XL size in DE-CT and PC-CT respectively. The complete ANOVA and Tukey pairwise comparison results are shown in Table 2, that is Figure 7.

The results of the analysis of variance indicate that phantom size, iodine concentration, phantom displacement from the iso-center, and solvent type (water or blood) are significant parameters for both scanners. However, the radiation dose only affects the DE-CT scanner and not the PC-CT scanner. Further analysis using Tukey pairwise comparison within the significant groups reveals that there is a significant difference in means between high (15 mg/mL) and lower (2 and 5 mg/mL) iodine concentrations in both scanners. This difference is more pronounced in the DE-CT scanner than in the PC-CT scanner (as shown in Figure 7a). Additionally, there were no significant differences in means obtained at extra-low, low, and standard doses for the IQ task in the PC-CT scanner (as shown in Figure 7c), as was the case for the VMI task. However, in the DE-CT scanner, measurements obtained at the standard dose were statistically different from the lower doses. The mean difference in the extra-large phantom size is significantly different from the medium and large sizes in both scanners (as shown in Figure 7d).

Based on these statistical results, the iodine size bias was characterized for each iodine concentration according to Equation (1) and is shown in Figure 5.

The IQ bias of −0.9 ± 0.15 was significantly lower in PC-CT compared to IB of 4.72 ± 0.22 in DE-CT.

## 4 ∣ DISCUSSION

Spectral CT scanners offer new opportunities in quantitative imaging through VMI and IQ. These methods are useful for a variety of clinical tasks, including tumor, staging, and tissue differentiation. To be considered fully quantitative they must be accurate, precise, and repeatable. Numerous studies on early-generation scanners have been conducted, testing the influence of many scan- and patient-related parameters on the accuracy and precision of VMI and IQ. Through their inherent ability to differentiate the energies of detected photons, paired with uniform spectral weighting and low electronic noise, a new generation of CT scanners with PCD offer new potential for spectral imaging. However, studies by Sartoretti et al.^18,26^ and Booij et al.^25^ found little or no difference in quantitative performance at doses comparable with DRLs, whereas Rajendran et al.^24^ and Decker et al.^27^ obtained statistically significant improvement in CNR at low dose range. Liu et al.^28^ performed a quantitative evaluation of VMI levels at low doses, stating that PC-CT outperforms DE-CT in given conditions. The purpose of this study was to compare the advantages of photon-counting versus energy-integrating detection in quantitative imaging with two representative clinical CT scanners under the influence of major patient-related factors such as patient size, radiation dose, patient positioning, iodine concentrations, and their dissolving environment at very low dose levels. Compared to other similar studies,^27,28^ in addition to VMI performance evaluation, we evaluated the IQ task and included more patient-related parameters and analyzed cases where quantitative imaging becomes extremely difficult such as in extremely obese patients at low doses.

Using the ANOVA we found that low-dose quantitative imaging was significantly affected by most patient-related parameters in both scanners. However, the radiation dose did not significantly affect the accuracy of quantitative imaging in the PC-CT scanner. The PC-CT scanner showed an obvious improvement in the IQ task, as iodine concentrations could be estimated with good accuracy (D = −0.30 ± 0.02 mg/mL) and small bias (−0.90 ± 0.15 mg/mL), while the accuracy of VMI was comparable at higher VMI levels (in agreement with previous studies) but degraded in the 40 keV virtual images. The VMI bias was higher and the iodine bias was lower in the PC-CT than in the DE-CT scanner in our study. For the IQ task, some of the results of this study can be compared to the findings of Sartoretti et al.^26^ For the identical phantom circumferences (M size phantoms in both studies) the dose levels (${\mathrm{CTDI}}_{vol}=15$, 10, and 5 mGy) were on average 1.7× higher than in our study. The reported iodine error in the PC-CT scanner (∣D∣ = 0.32 ± 0.39) was comparable to the one obtained in the PC-CT scanner in our study (0.30 ± 0.02 mg/mL), and in the DE-CT scanner (∣D∣ = 0.36 ± 0.31) the reduction of the dose severely affected the accuracy of IQ (1.57 ± 0.04 mg/mL) in our study. Iodine quantification bias results were compared to the largest study^13^ evaluating the most widespread dual-energy scanners from different vendors. The dose reported in their study was on average 1.6× higher for nearly matching phantom sizes (elliptical phantom 40×30 cm versus the L size phantom of diameter 40 cm) and the iodine inserts used in both studies were of the same concentration. The iodine bias range obtained from several dual-energy scanners (−2.6 to 1.5 mg/mL) was comparable to the iodine bias in PC-CT (−0.9 ± 0.15 mg/mL) but not with the bias in DE-CT (4.72 ± 0.22 mg/mL) scanner used in our study. Therefore, IQ at very low doses in PC-CT produces a comparable accuracy and bias to that of dual-energy scanners at DRLs, but the reduction of dose in the same DE-CT scanner type significantly increases the iodine bias. The comparisons with the previous studies further corroborate our result that radiation dose was not a statistically significant factor in the PC-CT scanner for the IQ task.

The size of the phantom was the most influential factor when estimating both quantitative maps. The size factor has been investigated before^31^ and some vendors have implemented a size-dependent calibration factor that re-scales the iodine concentrations based on the effective size of the patient.The main effects influencing the accuracy at increased phantom size are the combined effects of photon starvation and energy weighing of the signal inside the detector. Because photon starvation is energy-dependent (beam hardening effect), the improved spectral weighting of low-energy photons in PCD becomes even more important in the imaging of large and dense objects at low doses. The combined effect of beam hardening and sub-optimal energy weighting has a particular influence on IQ because the most prominent feature of iodine, its K-edge, is located at the low end of diagnostic energy spectra (33.2 keV). Indeed, the mean differences in the Tukey pairwise comparisons were the largest between low and high iodine concentrations and between medium and larger phantom sizes. For comparison, IQ accuracy for M and L size phantom is comparable in PC-CT (D = 0.47 ± 0.01 → D = 0.67 ± 0.03), while a significant drop in accuracy is observed in DE-CT for the two sizes (D = 0.50 ± 0.03 → D = 1.59 ± 0.06). However, when photon statistic becomes very low such as in XL size phantom, both scanners exhibit low IQ accuracy. Because the L and XL phantom sizes used in this study are representative of obese and severely obese patients and at the same time they make up nearly 42% and 10% of the US adult population (NHANES 2017–18), our results suggest improved quantitative imaging performance in PC-CT for the substantial part of US population.

The VMI task is different from the IQ task in that the basis materials used for material decomposition usually don’t contain a K-edge. An advantage of improved photon statistics at very low energies in the PC-CT scanner (due to improved weighting of low energy photons) that is crucial for the IQ task, seems to diminish in the VMI tasks for the condition of low-dose-obese patient imaging, especially for the estimation of bone VMI HU values and other dense tissues. Overall, the DE-CT scanner HU accuracy for all VMI levels (13 ± 2 HU) was within the range (11.4–52.0 HU) reported in prior studies,^13^ and compared to these values the PC-CT scanner showed similar accuracy (−28 ± 3 HU) but in opposite direction. Further investigation revealed that the significant negative bias (−83 ± 3 HU) was driven by an underestimation of 40 keV HU values in XL phantom (top-right Figure 3), particularly in high-concentration calcium and iodine inserts (see Appendix). Liu et al.^28^ reported larger deviations from the ground truth in 40 keV images compared to higher virtual monochromatic levels for both PC-CT at 120 kVp and DE-CT at 100/150Sn kVp scanner. Since we used much larger patient sizes (L and XL) and denser inserts (calcium 100 and 300 mg/mL and iodine 15 mg/mL),the accuracy of estimating HU values at 40 keV was significantly reduced in PC-CT. Previous studies found that an increase in tube potentials in DE-CT benefits VMI at low doses, despite decreased spectral separation and decreased attenuation difference between basis materials. Perhaps, following the same logic, PC-CT scanners could benefit from increased tube potentials and/or threshold optimization for the specific case.

The analysis of the influence of the dissolving environment on the accuracy of iodine estimation in the two special inserts of 2 mg/mL with different body fluids backgrounds showed statistically significant differences in both scanners. The insert containing blood produced consistently higher iodine concentration measurements and positive iodine bias in both scanners. This is because blood contains a certain amount of iron, which can erroneously mimic iodine-induced attenuation. Thus, it is worth noting that the true iodine concentration in blood could be slightly lower compared to the measured values. Lastly, the offset of 5 cm from the iso-center didn’t cause any differences in VMI performance in the PC-CT scanner, but it was significant for iodine imaging and for both quantitative tasks in the DE-CT.

Some of the limitations of our study should be noted. We performed this study on phantoms, thus, results may slightly differ in actual patients. Our study covered a wide range of patient-related parameters relative to clinical practice but we mostly focused on low-dose performance. Although we had different levels of iodine concentration, we didn’t estimate a minimal detectable concentration difference^14^ due to the lack of suitable inserts. Although expected to be minimal, some differences in results could exist due to the difference in smoothing kernel implementation and reconstruction software version.

## 5 ∣ CONCLUSIONS

According to our statistical analysis, PC-CT has the potential to achieve better quantitative performance at lower radiation doses. Our study found that the Siemens NAEOTOM Alpha PC-CT scanner showed comparable accuracy in iodine and VMI imaging between low and standard radiation dose levels. In contrast, the DE-CT scanner’s performance was affected by radiation dose levels and showed reduced accuracy at lower radiation doses. The PC-CT scanner outperforms the DE-CT scanner in the IQ task in all cases. The accuracy of VMI is comparable between scanners for normal and obese patients, but in extreme cases of very large patients and dense material inserts, DE-CT seems to benefit from the increased tube potential configurations available on the system, outperforming the PC-CT scanner.

## Figures and Tables

**TABLE 1:** Scan parameters of both scanners.

Scanner model	Phantom size	${\mathrm{CTDI}}_{vol}$ (mGy)	Offset (cm)	Tube voltage (kVp)	Tube current (mAs)
**Somatom FORCE**	M	2.6, 5.2, 10.4	0;5	80/150Sn; 100/150Sn	18, 36, 73
	L	6.8, 13.6, 27.2	0;5	80/150Sn;100/150Sn	48, 95, 190
	XL	20, 40, 70	0;5	80/150Sn;100/150Sn	140, 280, 557
**NAEOTOM Alpha**	M	2.6, 5.2, 10.4	0;5	120; 140	19, 36, 73
	L	6.8, 13.6, 27.2	0;5	120; 140	43, 68, 172
	XL	20, 40, 70	0;5	120; 140	127, 253, 380

**TABLE 2:** Analysis of variance.

	VMI		IQ	
	EID	PCD	EID	PCD
Quantitative task	19.15***	505.63***	-	-
Phantom size	288.64***	499.05***	220.61***	1934***
${\mathrm{CTDI}}_{vol}$	21.22***	0.61	5.35**	1.70
Material type	115.05***	77.42***	223.73***	294.34***
Displacement from iso-center	52.73***	0.22	15.68***	44.88***
Solvent	-	-	20.23***	9.17**

Abbreviations: EID, energy-integrating detectors; IQ, Iodine quantification; PCD, photon-counting detectors; VMI, Virtual monochromatic imaging.

*** *p* < 0

** *p* < 0.001

* *p* < 0.01;

⋅ *p* < 0.05.

**FIGURE A1:**
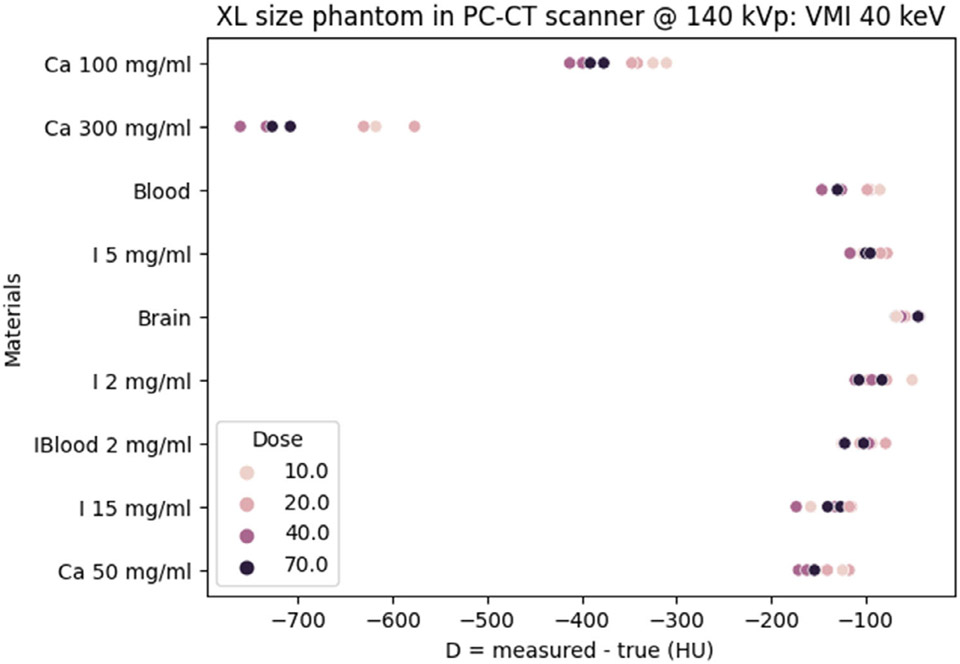
The accuracy of VMI 40 keV HU values in the PC-CT scanner for XL phantom size. Measurements were repeated twice at doses of 10, 20, 40, and 70 mGy. HU, Hounsfield units; PC-CT, photon-counting CT; VMI, Virtual monochromatic imaging.

## APPENDIX A

The PC-CT scanner is underestimating 40 keV virtual monochromatic levels in the extra-large phantom, particularly in higher-density inserts. This underestimation remains consistent even with increased doses, indicating that low VMIs may not be accurate in extremely obese patients, particularly for dense tissues. The results for the particular case with repeated scans for each dose are given in Figure A1.

## References

[1] Vrbaski S, Longo R, Contillo A. From spectral decomposition through SVD to quantitative description of monochromatic CT images: a phantom study. Med Imaging 2022: Phys Med Imaging. 2022;12031:1203132.

[2] Albrecht MH, Vogl TJ, Martin SS, Review of clinical applications for virtual monoenergetic dual-energy CT. Radiology. 2019;293(2):260–271. 31502938 10.1148/radiol.2019182297

[3] Rassouli N, Chalian H, Rajiah P, Dhanantwari A, Landeras L. Assessment of 70-keV virtual monoenergetic spectral images in abdominal CT imaging: a comparison study to conventional polychromatic 120-kVp images. Abdom Radiol (NY). 2017;42(10):2579–2586. 28421243 10.1007/s00261-017-1151-2

[4] Kaup M, Scholtz JE, Engler A, Dual-energy computed tomography virtual monoenergetic imaging of lung cancer: assessment of optimal energy levels. J Comput Assist Tomogr. 2016;40(1):80–85. 26466115 10.1097/RCT.0000000000000319

[5] Neuhaus V, Große Hokamp N, Abdullayev N, Comparison of virtual monoenergetic and polyenergetic images reconstructed from dual-layer detector CT angiography of the head and neck. Eur Radiol. 2018;28(3):1102–1110. 29018958 10.1007/s00330-017-5081-8

[6] Chang S, Hur J, Im DJ, Dual-energy CT-based iodine quantification for differentiating pulmonary artery sarcoma from pulmonary thromboembolism: a pilot study. Eur Radiol. 2016;26(9):3162–3170. 26638163 10.1007/s00330-015-4140-2

[7] Pelgrim GJ, van Hamersvelt RW, Willemink MJ, Accuracy of iodine quantification using dual energy CT in latest generation dual source and dual layer CT. Eur Radiol. 2017;27(9):3904–3912. 28168368 10.1007/s00330-017-4752-9 PMC5544802

[8] Yan WQ, Xin YK, Jing Y, Iodine quantification using dual-energy computed tomography for differentiating thymic tumors. J Comput Assist Tomogr. 2018;42(6):873–880. 30339550 10.1097/RCT.0000000000000800 PMC6250292

[9] Rizzo S, Radice D, Femia M, Metastatic and non-metastatic lymph nodes: quantification and different distribution of iodine uptake assessed by dual-energy CT. Eur Radiol. 2018;28(2):760–769. 28835993 10.1007/s00330-017-5015-5

[10] Mileto A, Marin D, Ramirez-Giraldo JC, Accuracy of contrast-enhanced dual-energy MDCT for the assessment of iodine uptake in renal lesions. Am J Roentgenology. 2014;202(5):W466–W474. American Roentgen Ray Society. 10.2214/AJR.13.11450 24758682

[11] Marin D, Davis D, Roy Choudhury K, Characterization of small focal renal lesions: diagnostic accuracy with single-phase contrast-enhanced dual-energy CT with material attenuation analysis compared with conventional attenuation measurements. Radiology. 2017;284(3):737–747. 28353408 10.1148/radiol.2017161872

[12] Martin SS, Weidinger S, Czwikla R, Iodine and fat quantification for differentiation of adrenal gland adenomas from metastases using third-generation dual-source dual-energy computed tomography. Invest Radiol. 2018;53(3):173–178. 28990974 10.1097/RLI.0000000000000425

[13] Jacobsen MC, Schellingerhout D, Wood CA, Intermanufacturer comparison of dual-energy CT iodine quantification and monochromatic attenuation: A phantom study. Radiology. 2018;287(1):224–234. Radiological Society of North America. 29185902 10.1148/radiol.2017170896

[14] Euler A, Solomon J, Mazurowski MA, Samei E, Nelson RC. How accurate and precise are CT based measurements of iodine concentration? A comparison of the minimum detectable concentration difference among single source and dual source dual energy CT in a phantom study. Eur Radiol. 2019;29(4):2069–2078. 30276672 10.1007/s00330-018-5736-0

[15] Sauter AP, Kopp FK, Münzel D, Accuracy of iodine quantification in dual-layer spectral CT: influence of iterative reconstruction,patient habitus and tube parameters. Eur J Radiol. 2018;102:83–88. 29685549 10.1016/j.ejrad.2018.03.009

[16] Harsaker V, Jensen K, Andersen HK, Martinsen AC. Quantitative benchmarking of iodine imaging for two CT spectral imaging technologies: a phantom study. Eur Radiol Exper. 2021;5(1):24. 34159477 10.1186/s41747-021-00224-2 PMC8219825

[17] Feuerlein S, Heye TJ, Bashir MR, Boll DT. Iodine quantification using dual-energy multidetector computed tomography imaging: phantom study assessing the impact of iterative reconstruction schemes and patient habitus on accuracy. Invest Radiol. 2012;47(11):656–661. 22996313 10.1097/RLI.0b013e31826585bb

[18] Sartoretti T, Landsmann A, Nakhostin D, Quantum iterative reconstruction for abdominal photon-counting detector CT improves image quality. Radiology. 2022;303(2):339–348. Radiological Society of North America. 35103540 10.1148/radiol.211931

[19] Willemink MJ, Persson M, Pourmorteza A, Pelc NJ, Fleischmann D. Photon-counting CT: technical principles and clinical prospects. Radiology. 2018;289(2):293–312. 30179101 10.1148/radiol.2018172656

[20] Danielsson M, Persson M, Sjölin M. Photon-counting X-ray detectors for CT. Phys Med Biol. 2021;66(3):03TR01. IOP Publishing. 10.1088/1361-6560/abc5a5 33113525

[21] Si-Mohamed S, Bar-Ness D, Sigovan M, Multicolour imaging with spectral photon-counting CT: a phantom study. Eur Radiol Exper. 2018;2(1):34. 30327898 10.1186/s41747-018-0063-4 PMC6191405

[22] Tao A, Huang R, Tao S, Michalak GJ, McCollough CH, Leng S. Dual-source photon counting detector CT with a tin filter: a phantom study on iodine quantification performance. Phys Med Biol. 2019;64(11):115 019. 10.1088/1361-6560/ab1c34 PMC6598702 31018197

[23] Farhadi F, Rajagopal JR, Nikpanah M, Review of technical advancements and clinical applications of photon-counting computed tomography in imaging of the thorax. J Thorac Imaging. 2021;36(2):84–94. 33399350 10.1097/RTI.0000000000000569

[24] Rajendran K, Petersilka M, Henning A, First clinical photon-counting detector CT system: technical evaluation. Radiology. 2022;303(1):130–138. Radiological Society of North America. 34904876 10.1148/radiol.212579 PMC8940675

[25] Booij R, van der Werf NR, Dijkshoorn ML, van der Lugt A, van Straten M. Assessment of iodine contrast-to-noise ratio in virtual monoenergetic images reconstructed from dual-source energy-integrating CT and photon-counting CT data. Diagnostics. 2022;12(6):1467. number: 6 Multidisciplinary Digital Publishing Institute. 35741277 10.3390/diagnostics12061467 PMC9222007

[26] Sartoretti T, Mergen V, Jungblut L, Alkadhi H, Euler A. Liver iodine quantification with photon-counting detector CT: accuracy in an abdominal phantom and feasibility in patients. Acad Radiol. 2022;30(3):461–469. 35644755 10.1016/j.acra.2022.04.021

[27] Decker JA, Bette S, Lubina N, Low-dose CT of the abdomen: initial experience on a novel photon-counting detector CT and comparison with energy-integrating detector CT. Eur J Radiol. 2022;148:110 181. 10.1016/j.ejrad.2022.110181 35121331

[28] Liu LP, Shapira N, Chen AA, First-generation clinical dual-source photon-counting CT: ultra-low-dose quantitative spectral imaging. Eur Radiol. 2022;32(12):8579–8587. 35708838 10.1007/s00330-022-08933-x PMC10071880

[29] Gutjahr R, Halaweish AF, Yu Z, Human imaging with photon-counting-based CT at clinical dose levels: contrast-to-noise ratio and cadaver studies. Invest Radiol. 2016;51(7):421–429. 26818529 10.1097/RLI.0000000000000251 PMC4899181

[30] Sawall S, Klein L, Wehrse E, Threshold-dependent iodine imaging and spectral separation in a whole-body photon-counting CT system. Eur Radiol. 2021;31(9):6631–6639. 33713171 10.1007/s00330-021-07786-0 PMC8379121

[31] Koonce JD, Vliegenthart R, Schoepf UJ, Accuracy of dual-energy computed tomography for the measurement of iodine concentration using cardiac CT protocols: validation in a phantom model. Eur Radiol. 2014;24(2):512–518. 24141716 10.1007/s00330-013-3040-6 PMC4126413

